# Oenological Characteristics of Selected *Saccharomyces* and Non-*Saccharomyces* Isolates Obtained from Polish Grape Wines of Spontaneous Fermentation and Their Potential as Wine Starter Cultures

**DOI:** 10.3390/molecules31081274

**Published:** 2026-04-13

**Authors:** Monika Kordowska-Wiater, Anna Stój, Elwira Komoń-Janczara, Monika Pytka, Adam Staniszewski, Magdalena Walasek, Tomasz Czernecki, Magdalena Kapłan

**Affiliations:** 1 Department of Biotechnology, Microbiology and Human Nutrition, University of Life Sciences in Lublin, Skromna 8, 20-704 Lublin, Poland; anna.stoj@up.lublin.pl (A.S.); elwira.komon.janczara@up.lublin.pl (E.K.-J.); monika.pytka@up.lublin.pl (M.P.); magda.walasek86@gmail.com (M.W.); tomasz.czernecki@up.lublin.pl (T.C.); 2 Department of Invertebrate Ecophysiology and Experimental Biology, University of Life Sciences in Lublin, Doświadczalna 50a, 20-280 Lublin, Poland; adam.staniszewski@up.lublin.pl; 3 Institute of Horticulture Production, University of Life Sciences in Lublin, Głęboka 28, 20-612 Lublin, Poland; magdalena.kaplan@up.lublin.pl

**Keywords:** *Saccharomyces*, non-*Saccharomyces*, oenological features, wine yeasts, ethanolic fermentation

## Abstract

Spontaneously fermented wines are a habitat for many *Saccharomyces* and non-*Saccharomyces* strains that are typical for a given region. The isolates obtained can serve as regional starter cultures for winemaking. The aim of this study was to isolate, identify and evaluate the oenological properties and fermentation suitability of selected yeast isolates obtained from Polish spontaneously fermented grape wines. The isolated yeasts were genetically identified and characterised in terms of ethanol tolerance, enzymatic activities, H_2_S production, and preliminary killer activity. In small-scale fermentations conducted in CDGJ medium and grape juice, the fermentation rate, pH, number of yeast, content of sugars, ethanol, organic acids and volatile compounds were determined. Genetic identification revealed the species: *Saccharomyces cerevisiae*, *S. paradoxus*, *Metschnikowia pulcherrima*, *M. ziziphicola*, *Hanseniaspora uvarum*, and *Pichia kluyveri*. Non-*Saccharomyces* and *Saccharomyces* strains grew poorly in the presence of 4–6% (*v*/*v*) and 14–16% (*v*/*v*) ethanol, respectively. The yeasts had varied enzymatic activities in API ZYM tests, and production of H_2_S, but did not exhibit killer activity. The monocultures showed differences in fermentation rates. The best growth was recorded for all strains during grape juice fermentation, up to 10^9^ cfu/mL, producing ethanol and glycerol in the range of 53.92–86.54 g/L and 0.0–4.48 g/L. Yeasts produced characteristic volatile compounds, e.g., esters: 2-phenylethyl acetate and ethyl decanoate. The monocultures of isolated yeasts can be used in fermentation of grape must, yielding wines with diverse characteristics in terms of ethanol, organic acids and volatile compounds.

## 1. Introduction

Yeasts colonise vineyard environments and are an important part of grape microbiota. They are usually divided into two groups: *Saccharomyces* and non-*Saccharomyces*. Grapes are the main source of non-*Saccharomyces* yeasts, which can be divided into three groups. The first one includes aerobic yeast belonging to *Pichia* ssp., *Debaryomyces* ssp., *Rhodotorula* ssp., *Candida* ssp. and *Cryptococcus albidus*. The second one is characterised by yeasts with low fermentation activity, e.g., *Kloeckera apiculata*, *K. apis* and *K. javanica*. The third group includes the fermenting strains, such as *Kluyveromyces marxianus*, *Torulaspora* ssp., and *Zygosaccharomyces* ssp. [1,2]. Other researchers mention the following types and species: *Hanseniaspora* (*H. vineae*, *H. uvarum*), *Pichia* (*P. fermentans*), *Metschnikowia* (*M. pulcherrima*), *Starmerella* (*S. bacillaris*, *S. stellata*), *Torulaspora delbrueckii*, *Schizosaccharomyces pombe*, *Lachancea thermotolerans*, *Kazachstania aerobia*, and others [3,4]. Interestingly, *Saccharomyces* yeasts are not the dominant type on the surface of grapes. Yeasts are the key drivers of fermentation in winemaking, transforming sugar into alcohol and shaping the wine’s aroma, flavour, and texture. In addition to ethanol, these microorganisms are responsible for the production of various metabolites (other alcohols, glycerol, organic acids, phenolic compounds, aromatic substances, and other products) and the secretion of enzymes (esterases, glucosidases, lipases, and proteases) that are important for the sensory characteristics of wines. Some yeasts can also reduce the concentrations of unwanted compounds, such as ochratoxin A, ethyl carbamate, and biogenic amines [5,6].

Naturally occurring non-*Saccharomyces* and *Saccharomyces* yeasts are involved in the spontaneous fermentation of must or grape juice. This process is composed of the sequential development of various species, which are involved in the conversion of complex compounds (primarily carbohydrates) into simpler ones with the simultaneous production of various beneficial metabolites [7]. The process depends not only on endogenous microbiota, but also on the grape variety, climatic conditions and fermentation parameters. During fermentation, there is a succession of representatives of the genera *Hanseniaspora* (anamorth *Kloeckera*), *Metschnikowia*, *Pichia*, *Candida*, and finally *Saccharomyces* dominates, which leads to the development of the characteristic features of wine. In the early stages of fermentation (the first 3–5 days), the number of non-*Saccharomyces* yeasts ranges from 10^3^ to 10^5^ cfu/mL to 10^6^–10^7^ cfu/mL [8,9,10]. However, the abundance of *Saccharomyces* yeast, although dominant especially at the end of fermentation, occurs at different times, depending mainly on the non-*Saccharomyces*/*S. cerevisiae* ratio and the properties of yeast species constituting the non-*Saccharomyces* population [11,12]. Spontaneous fermentation allows for the production of wines characteristic of a given region thanks to unique grape varieties, but on the other hand, it carries the risk of slowing down or stopping fermentation, or the proliferation of undesirable yeasts that deteriorate the taste and aroma through increased production of acetic acid, ethyl acetate or acetoin [13]. Another option is controlled fermentation with the use of *S. cerevisiae* or non-*Saccharomyces* starter monocultures, which can help to enhance particular and specific characteristics of a wine, monitor microbial populations, and limit the growth of spoilage yeasts [14]. There is currently considerable interest in isolating and characterising specific strains that can be used as starter monocultures to help achieve wine flavour diversity while reducing the risk of spoilage. An added benefit may be linking characteristic regional yeast populations to the sensory differences found in wines from different geographic regions [3]. Some non-*Saccharomyces* yeasts have already been validated as starter cultures, while many are still being investigated [15].

The oenological characteristics of yeast include a set of features that are important in wine production, such as the following: carbohydrate fermentation capacity, ethanol and SO_2_ tolerance, and the production of secondary metabolites and aromatic compounds. A thorough understanding of isolates obtained from grapes or winemaking environments helps to select the most suitable strains as starter cultures, the addition of which could direct the fermentation process towards obtaining wine with the desired characteristics. Starter cultures based on Polish strains would be desirable in the context of the constantly developing wine industry in Poland. The improvement in climatic conditions in Poland has considerably boosted interest in viticulture and wine production in the last decade. The number of vineyards in Poland in 2024/25 was 703, the number of wine producers was 504, the area under vines increased to 1068.49 ha, and wine production increased to 21,423.64 hl [16]. Despite growing interest in winemaking in Poland, little research has been conducted on isolating and characterising the biodiversity of native yeast strains adapted to Poland’s cool climate [17,18,19,20].

The aim of the study was to isolate, identify and characterise selected yeasts obtained from spontaneously fermented wines made from Polish Regent grapes originating from three vineyards in the Małopolska Vistula River Gorge. This comprehensive study covered both biochemical and physiological characteristics as well as the fermentation capabilities of pre-selected *Saccharomyces* and non-*Saccharomyces* isolates under controlled small-scale fermentation conditions. The results obtained are a valuable aid in the development of new starter cultures dedicated to the production of Polish wines.

## 2. Results and Discussion

### 2.1. Isolation and Genetic Identification of Yeast Strains

During the isolation procedure on YGC agar and WLN agar, 44 types of colonies showing differences in their morphology were obtained from samples of wines produced during the spontaneous fermentation of Regent variety grapes. This WLN medium is specially dedicated to the visual distinguishing of non-*Saccharomyces* and *Saccharomyces* yeasts, and it has a specific application in winemaking for the isolation and preliminary identification of yeasts [6,21]. Microorganisms in all colonies were confirmed microscopically as yeasts. All isolates were assigned to individual species using Sanger sequencing of ITS1-5.8S rDNA-ITS2 regions. Comparison of the sequences with the GenBank database revealed that the isolates belong to five genera: *Saccharomyces*, *Metschnikowia*, *Pichia*, *Hanseniaspora*, and *Starmerella*, as is shown in Table 1, together with the number of isolates obtained, and morphological characteristics of their colonies on WLN Agar. There are many reports in scientific literature on the isolation of yeasts from spontaneously fermented wines made from local grape varieties. Some species correspond to those isolated in our study, e.g., Cioch-Skoneczny et al. [1] isolated strains belonged to the species *H. uvarum*, *Z. meyerae*, *M. pulcherrima*, *C. oleophila*, *C. railenensis*, *N. ishiwadae*, *P. membranifaciens*, *K. lactis*, and *S. cerevisiae* from wines of Regent and Rondo varieties obtained from Polish vineyards. Skotniczny et al. [22] isolated strains belonging to *H. uvarum*, *Metschnikowia* sp., *P. kluyveri*, and *S. cerevisiae*, among others, from plum musts originating in Poland, which confirms that these microorganisms are present in Polish orchards and vineyards and can be considered part of the typical fruit microbiota. Some isolates belonging to the same species were obtained from wines in Slovakia, which further confirms the preference of these species for the European region and climate [23,24]. On the other hand, Raymond Eder et al. [21] isolated *H. uvarum*, *S. bacillaris*, *P. kluyveri*, and *M. pulcherrima* from the initial phase, and *S. cerevisiae* strains from the later phase of fermentation of Malbec and Isabella musts in Argentina.

### 2.2. Ethanol Tolerance

**Figure 1:**
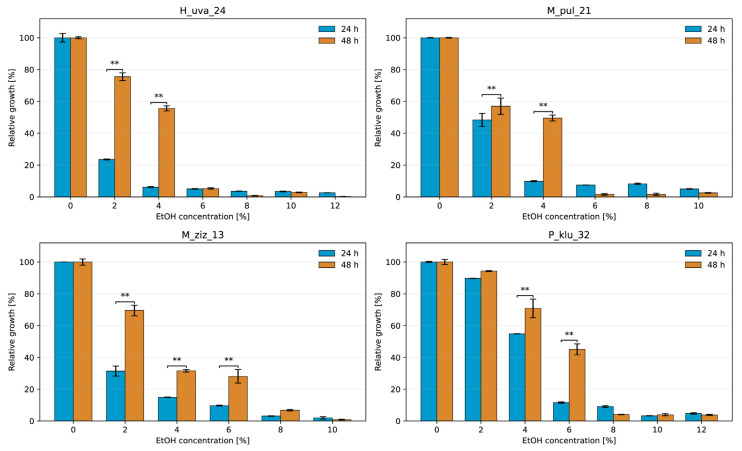
Relative growth of H_uva_24, M_pul_21, M_ziz_13, and P_klu_32 at different ethanol concentrations after 24 h and 48 h of incubation. Data are presented as mean ± SD and expressed as a percentage of the control. Significant differences between 24 h and 48 h at the same ethanol concentration are indicated by ** (Tukey’s test, α = 0.01).

**Figure 2:**
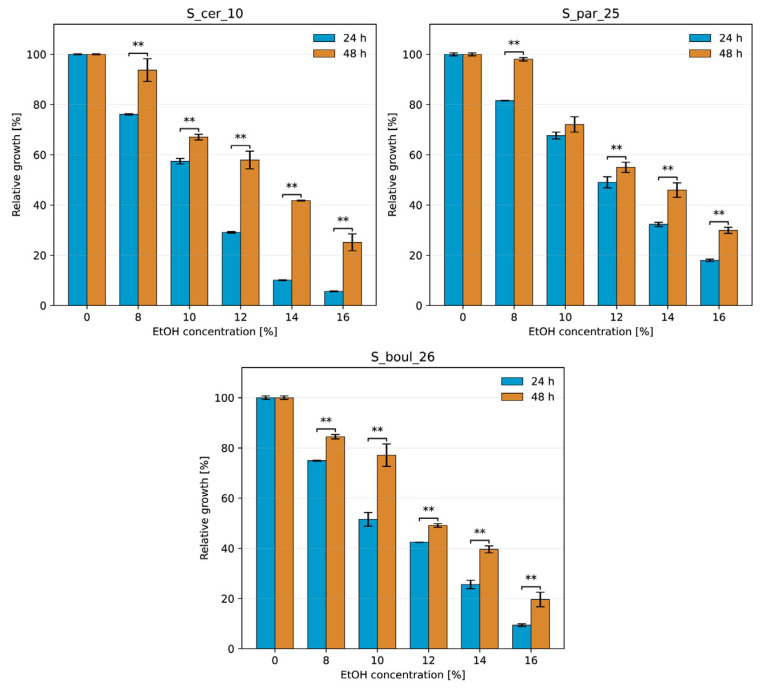
Relative growth of S_cer_10, S_par_25, and S_boul_26 at different ethanol concentrations after 24 h and 48 h of incubation. Data are presented as mean ± SD and expressed as a percentage of the control. Significant differences between 24 h and 48 h at the same ethanol concentration are indicated by ** (Tukey’s test, α = 0.01).

The relative growth of yeasts in the presence of various concentrations of added ethanol is shown in Figure 1 and Figure 2 as the percentage of control growth without stressor after 1 and 2 days of incubation, together with statistical analysis (Supplementary S1). *Saccharomyces* strains showed high relative growth in the presence of ethanol at concentrations of 8–10% (*v*/*v*), 84.5–98.05%, and 67–77% after 2 days of incubation. At higher ethanol concentrations, growth activity finally dropped to 19.6–29.9% in the presence of 16% (*v*/*v*) alcohol. The results indicate that the yeasts were able to multiply throughout the incubation period. These results are consistent with the literature [25,26,27]. Non-*Saccharomyces* yeasts confirmed their sensitivity and showed very low relative growth of a few percent at 8–12% (*v*/*v*) ethanol. The most resistant in this group was the Pichia strain, which showed 45% growth in the presence of 6% *v*/*v* ethanol after 2 days and only showed a sharp decline in numbers at 8% (*v*/*v*) ethanol addition. The very limited ethanol tolerance of non-*Saccharomyces* yeasts is in line with their known role as early-phase fermenters in wine and cider [28,29]. The percentage values of relative growth of *Metschnikowia* confirm the observation that these yeasts survive in 3–5% (*v*/*v*) ethanol and are important in co-fermentation [30,31,32].

### 2.3. Enzymatic Activity of Yeasts

The results of the API ZYM test presented in Table 2 indicate differences in the enzymatic activities of yeast isolates from wines, which may be an important indicator of their technological potential, a specific functional ‘fingerprint’ of the strain. Particularly important from an oenological perspective is the presence of β-glucosidase and α-glucosidase activity, enzymes involved in the hydrolysis of plant glycosides, leading respectively to the release of free aromatic compounds (e.g., terpenes) and increased availability of fermentable substrates. This phenomenon is important in the context of shaping the aromatic profile of wines (the development of fruit and floral notes), where some of the aroma precursors occur in a bound form. Particularly, many studies have focused on this ability in non-*Saccharomyces* yeasts, as described in the review article by Padilla et al. [33]. In the studies conducted, high and medium β-glucosidase activity was demonstrated by yeasts of the genus Metschnikowia: M_pul_21, M_ziz_13 and Pichia–P_klu_32, while α-glucosidase was produced by Metschnikowia, Pichia and S_boul_26 yeasts. At the same time, the activity of proteolytic and peptidolytic enzymes, such as leucine arylamidase and valine arylamidase, may indicate the ability of yeast to release amino acids from peptides present in must, as they are both a source of nitrogen for cells and substrates for the formation of higher alcohols and esters. Consequently, strains exhibiting higher aminopeptidase activity may potentially contribute to the intensification of fermentation aromas and better adaptation to stressful fermentation conditions. All of the strains tested had high levels of these enzyme activities, with the exception of H_uva_24. Studies based on API ZYM have shown that these enzyme activities are among the most commonly observed in yeasts isolated from wines, suggesting their role in adaptation to the must environment and the course of alcoholic fermentation [20]. The presence of esterase (C4) and esterase–lipase (C8) activity may be important in the context of ester conversion and lipid metabolism, which translates into the development of fermentation aroma and the sensory structure of wine. With the exception of H_uva_24, the yeasts studied in this work also produced these enzymes, with a tendency towards esterase activity predominance (C4). In turn, phosphatase activity demonstrated in all tested strains (particularly high in *Saccharomyces* and Pichia yeasts) may indicate metabolic adaptation to the acidic conditions typical of grape must and the ability to mobilise inorganic phosphates important for promoting yeast survival and fermentation stability. The API ZYM test shows differences between the enzymatic capabilities of S. cerevisiae and non-*Saccharomyces* yeasts, which usually exhibit a wider range of hydrolytic activities, which may translate into their greater involvement in the biotransformation of must compounds and the formation of the aromatic profile of wine, as well as the overall adaptation of strains to the must environment. At the same time, it was observed that no strain presented activity of lipase (C14), trypsin, chymotrypsin, α-galactosidase, β-galactosidase, β-glucuronidase, N-acetyl-β-glucosaminidase, and α –fucosidase. The application of this method to the enzymatic characterisation of wine yeasts revealed that the differences in glycosidases and arylamidases activities between *Metschnikowia*, *Hanseniaspora*, *Pichia*, and *Saccharomyces* strains were observed, but many of them were similar to the results presented here [20]. In studies by Tofalo et al. [34], *H. uvarum* and *Pichia* sp. strains generally did not produce α- and β- glucosidase, but differed in esterase, lipase–esterase, and alkaline phosphatase activities. *M. fructicola* showed high β-glucosidase activity and no α-glucosidase or phosphatase activity, with weak esterase and lipase–esterase activity. Mendoza et al. [35] showed that non-*Saccharomyces* species such as *M. pulcherrima* and *H. uvarum* showed higher β-glucosidase and esterase activities than *S. cerevisiae*. Morata et al. [32] also confirmed the ability of *Metchnikowia* strains to produce β-glucosidase. On the other hand, the results for *S. cerevisiae* strains obtained by Liszkowska et al. [36] were similar to our three *Saccharomyces* isolates in terms of phosphatase, esterase, lipase–esterase, arylamidase and glucosidase activity, demonstrating the consistency of the enzymatic predispositions of yeasts belonging to this genus. The pectolytic activity of yeast is an important technological feature in winemaking, as the enzymatic degradation of grape cell wall pectins reduces must viscosity, facilitates clarification, and improves filtration and colloidal stability of wine [37]. Since the API ZYM test does not directly cover the activity of key pectolytic enzymes, this determination was performed separately to complete the assessment of the overall potential for cell wall component degradation and the strain’s ability to biotransform the grape must matrix. Unfortunately, none of the strains tested yielded a positive result. In the literature, S. cerevisiae does not usually have this activity, but some Metschnikowia strains showed these activities [38,39,40].

### 2.4. H_2_S Production

The ability of yeast to produce H_2_S is an undesirable trait, as this compound negatively affects the aroma of wine—the so-called “rotten egg odour”—and lowers its sensory rating. It is known that different strains have significantly different predispositions to produce this compound; therefore, the selection of yeasts with low H_2_S production is an important quality feature. It has been found that approximately 1% of naturally occurring strains do not have this ability, and *S. cerevisiae* is usually responsible for the undesirable odour derived from H_2_S [41]. H_2_S production by microorganisms is indicated on BIGGY agar by the colouration of the colonies from brown to black. The strain with the highest ability to produce H_2_S was *P. kluyveri*, because the colour change in the colonies to brown–black was observed, while no production was observed in the case of *H. uvarum* and *M. pulcherrima*. The remaining strains showed a light (M_ziz_13) or medium (S_cer_10, S_ par_25, S_boul_26) brown coloration. Similar results for *H. uvarum* and *S. cerevisiae* strains were obtained by Staniszewski et al. [27], and for *H. uvarum*, where 88.5% did not produce this compound in the study of Polizzotto et al. [6]. In another study, most strains produced low or moderate levels of H_2_S and only 11% isolates, including some *H. uvarum* and *S. cerevisiae*, did not produce this detrimental compound [35]. In Raymond Eder et al. [21], studies 25% isolates of *H. uvarum* and *S. cerevisiae* from spontaneously fermented musts were not able to produce H_2_S.

### 2.5. Killer Activity

The phenomenon of killer toxin secretion by yeasts has been extensively studied. These extracellular proteins or glycoproteins can be produced by both *Saccharomyces* and non-*Saccharomyces* yeasts, including *Hanseniaspora*, *Pichia* and *Rhodotorula*, originating from various natural environments, including wine. This ability is of great importance in winemaking, as cultures with this property can prevent infections and deterioration of wine quality by spoilage yeasts, but on the other hand, they can inhibit the fermentation process [42]. Under the experimental conditions, none of the tested strains showed killer activity against the reference strains, i.e., *Kluyveromyces lactis*, *Candida parapsilosis*, and *Candida freyschussii*. After incubation, no growth inhibition zones surrounded by a blue border were observed around the wells with the supernatants of the tested yeast isolates. In the study of Mendoza et al. [35], most of the non-*Saccharomyces* yeasts showed a sensitive phenotype; neutral and killer phenotypes were found, respectively, in 15% and 5% of strains. Only the strains *H. uvarum* HuM18 and *S. cerevisiae* BSc410 were capable of producing killer toxin. Other studies reported that the most non-*Saccharomyces* yeasts presented a neutral or sensitive phenotype [43].

### 2.6. Small-Scale Fermentations in CDGJ Medium and Grape Juice

#### 2.6.1. Fermentation Rate

**Figure 3:**
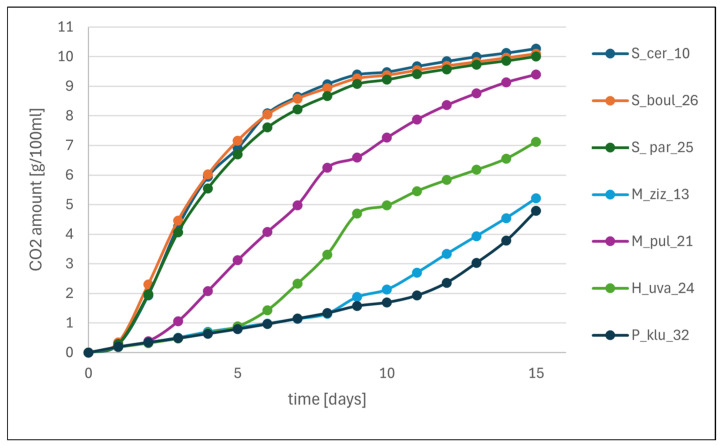
The fermentation rate of yeast monocultures on CDGJ medium is expressed as the amount of CO_2_ released at one-day intervals [means, *n* = 3].

Fermentation rate indicates how quickly yeasts begin and continue to ferment sugars and can realistically be used as starter cultures in winemaking, ensuring dynamic, consistent, and predictable fermentation, which is crucial when planning the process and predicting the quality of the final product. One of the basic parameters monitoring this process is the fermentation rate, determined by the amount of CO_2_ released, usually using the weighing method. In the first stage of vinification research, a synthetic CDGJ medium was used, which is designed to mimic grape juice and is used for preliminary studies [44]. Figure 3 shows the course of small-scale fermentations with the participation of monocultures of the tested yeasts. Three fermentation rates are clearly visible: (1) rapid fermentation for the first 6 days, followed by a gradual decline and then no change in CO_2_ levels, indicating the end of fermentation—this is a typical profile for *Saccharomyces* yeasts (S_cer_10, S_par_25, S_boul_26); (2) delayed fermentation—the amount of CO_2_ increased slowly with a spike on days 7–9, followed by a slowdown—this profile appeared in the M_pul_21 and H_uva_24 monocultures; (3) significantly delayed fermentation in the M_ziz_13 and P_klu_32 monocultures, where the increase in CO_2_ released was more noticeable from day 11 until the end of incubation. Similar results for *S. cerevisiae* were obtained by Raymond et al. [21] in test tube fermentations, where these yeasts produced approximately 6 g CO_2_/100 mL of medium, while they obtained significantly better fermentation efficiency for *H. uvarum* isolates.

**Figure 4:**
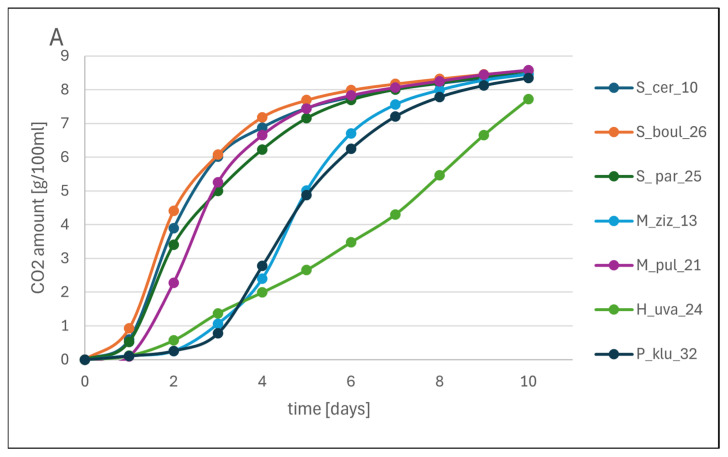

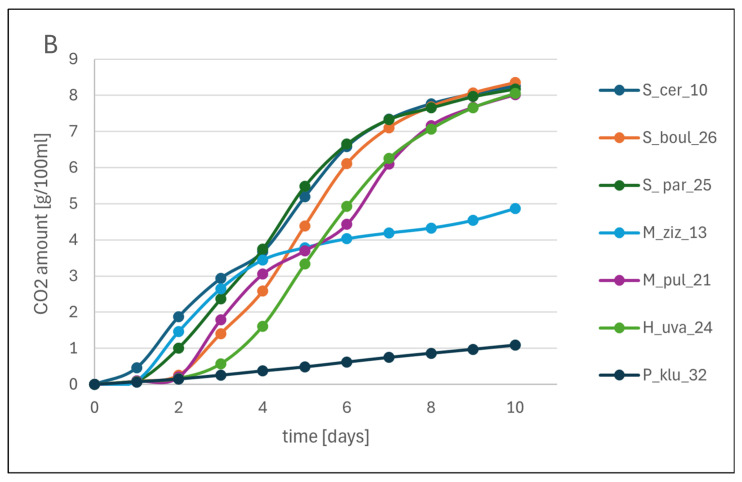
The fermentation rate of yeast monocultures on grape juice (**A**) and grape juice with SO_2_ (**B**) is expressed as the amount of CO_2_ released at one-day intervals [means, *n* = 3].

In small-scale fermentations carried out in grape juice, *Saccharomyces* yeasts exhibited a fermentation rate profile similar to that of the model medium, but the amount of CO_2_ released was finally lower by 1.2–1.4 g/100 mL. M_pul_21 yeast approached the fermentation rate of *Saccharomyces* and improved the result by 1 g CO_2_/100 mL over 10 days. The process dynamics improved in M_ziz_13 and P_klu_32, although it was delayed, but greater mass losses were observed from the third day onwards, while the weakest fermentation rate was observed in the H_uva_24 strain, but after 10 days, the amount of CO_2_ released increased by approx. 2.7 g/100 mL compared to the CDGJ medium. The addition of potassium metabisulphite slowed down the fermentation rate of all strains by 1–2 days, except for H_uva_24, which had a rate most similar to the fermentation of the juice itself, and P_klu_32, which was strongly inhibited. Detailed data are presented in Figure 4.

#### 2.6.2. The Number of Yeasts in Monocultures

The changes in the number of yeasts in CDGJ medium are consistent with the course of small-scale fermentation (Table 3), as a decrease in the number of *Saccharomyces* yeasts was observed relative to the initial number of 1.0–3.3 × 10^5^ cfu/mL, and a smaller or larger increase in the number of non-*Saccharomyces* yeasts, even up to 1.03–1.33 × 10^6^ cfu/mL at the end of the fermentation process. During grape juice fermentation, all strains showed higher growth (Table 4), and ultimately, the number of *Saccharomyces* ranged from 1.7 to 7.02 × 10^8^ cfu/mL, similar to Varela et al. [45], where *S. cerevisiae* reached a value of 1.1 × 10^8^ cfu/mL. Most non-*Saccharomyces* isolates reached a count above 10^9^ cfu/mL (except for M_pul_21—approx. 8 × 10^8^ cfu/mL). In juice with metabisulphite added, all strains exceeded 10^8^ cfu/mL, except for P_klu_32, whose growth was significantly inhibited, which was also correlated with a low fermentation rate. Researchers often perform a final biomass to determine the effect of fermentation conditions on the growth of strains tested for application purposes. For example, Mendoza et al. [35] determined the log cfu/mL count for many non-*Saccharomyces* strains studied, including *H. uvarum* in the range of 1.7–6.24 log cfu/mL with a predominance between 3 and 5 log cfu/mL, and *M. pulcherrima* strains reached 6.89–7. 13 log cfu/mL after 10 days of grape juice fermentation. These researchers also observed that *H. uvarum* strains had different sensitivities to SO_2_, and *M. pulcherrima* strains showed similar growth to *S. cerevisiae* in the presence of SO_2_, which is consistent with our observations and demonstrates the importance of this analysis in strain selection.

#### 2.6.3. Metabolic Activity of Yeast Strains

**Figure 5:**
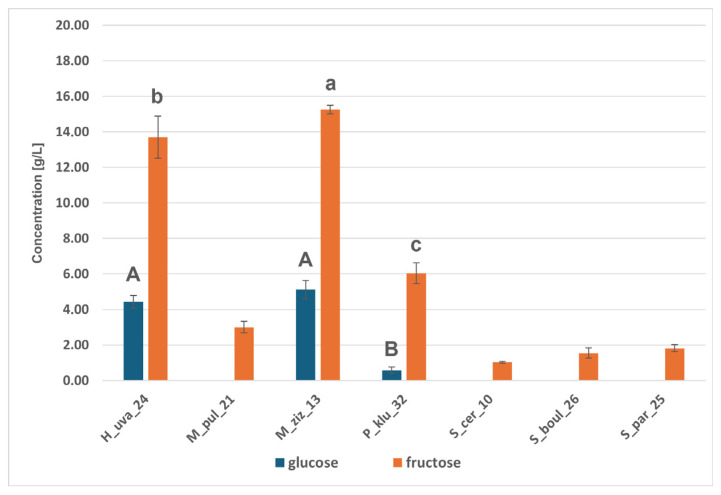
Residual glucose and fructose concentrations (g/L) after CGDMJ fermentation by the indicated yeast strains. Uppercase letters refer to statistical groupings for glucose, whereas lowercase letters refer to statistical groupings for fructose; bars sharing the same letter are not significantly different, while different letters indicate significant differences among strains (*p* < 0.01).

**Figure 6:**
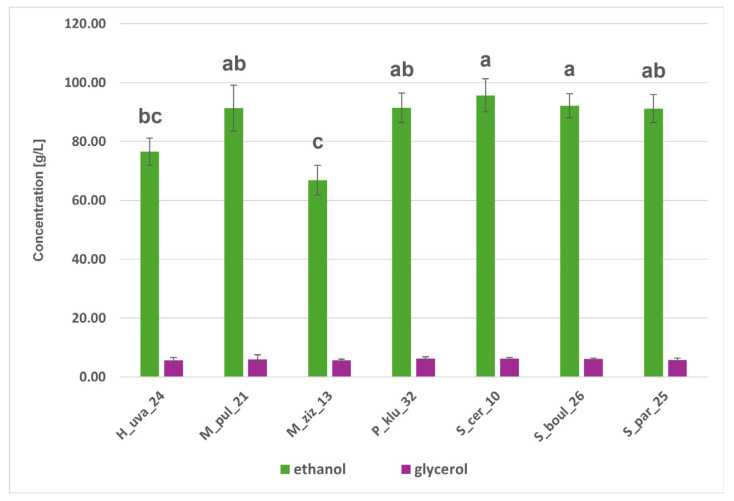
Means of ethanol and glycerol concentrations (g/L) produced in CGDMJ fermentation by the indicated yeast strains. Bars represent mean ± SD. Different letters denote statistically significant differences among strains (*p* < 0.01) for the marked parameter.

**Figure 7:**
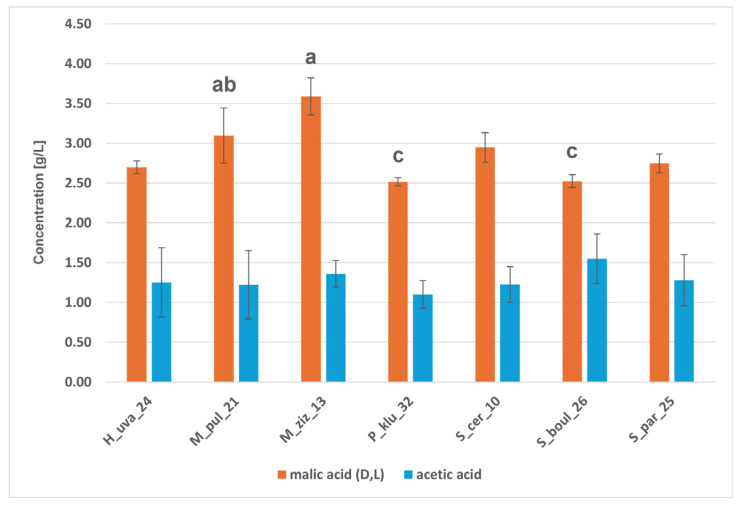
Means of malic acid (D,L) and acetic acid concentrations (g/L) in fermentations performed on CGDMJ by the indicated yeast strains. Bars represent mean ± SD. Different letters indicate statistically significant differences among strains (*p* < 0.01) within the respective compound.

The isolates studied were used as monocultures to carry out model fermentations in CDGJ medium with a strictly defined composition. Fermenting yeasts are capable of anaerobic utilisation of basic simple sugars such as glucose and fructose, which are present simultaneously in fruit juices and wine musts, and act as electron donors and acceptors and sources of carbon [46]. HPLC analysis revealed the ability to ferment glucose and fructose into ethanol, glycerol, and organic acids: acetic, malic, and citric (Figure 5, Figure 6 and Figure 7 and Supplementary S2). Figure 5 shows that the *Saccharomyces* strains utilised glucose completely, while fructose was assimilated less efficiently, with 4–5 g/L and 13–15 g/L of glucose and fructose remaining in the H_uva_24 and M_ziz_13 cultures, respectively. *Saccharomyces* yeasts produced over 90 g/L of ethanol, and among the non-*Saccharomyces* yeasts, M_pul_21 and P_klu_32 proved to be comparably good producers (approx. 90 g/L of ethanol). H_uva_24 and M_ziz_13 proved to be statistically significantly different from the above, producing 75 and 68 g/L of this alcohol, respectively (Figure 6). Ethanol is necessary for cells to maintain redox balance and produce ATP, which is essential for growth [46]. Ethanol is quantitatively the most important product of alcoholic fermentation and, at the same time, one of the key factors determining the physicochemical and sensory properties of wine. It affects the chemical equilibrium system, the solubility of compounds and the microbiological and oxidative stability of the product [47,48]. An important secondary metabolite is glycerol, which is often produced in response to osmotic stress and, in addition to its protective effect on cells, contributes to improving the viscosity of wine, giving it a smoother, softer and fuller taste [46,47]. All strains produced glycerol in comparable amounts (5.62–6.23 g/L) (Figure 6). Man-Hsi Lin et al. [49], conducting monoculture fermentations in CDGJM, obtained 110 g/L of ethanol and approx. 9 g/L of glycerol for *S. cerevisiae*, and between 40 and 80 g/L of ethanol and from a few to approx. 13 g/L of glycerol for non-*Saccharomyces*. The isolates studied were characterised by varying abilities to produce organic acids; they produced little acetic and citric acid. Malic acid in concentrations above 3 g/L appeared in the cultures of both *Metschnikowia* strains and the results for M_ziz_13–3.9 g/L and P_klu_32–2.5 g/L were significantly different) (Figure 7). The ability of certain yeast species, such as *S. cerevisiae*, to produce specific organic acids, including acetic, malic, and citric acids, is described in the scientific literature [50,51].

**Figure 8:**
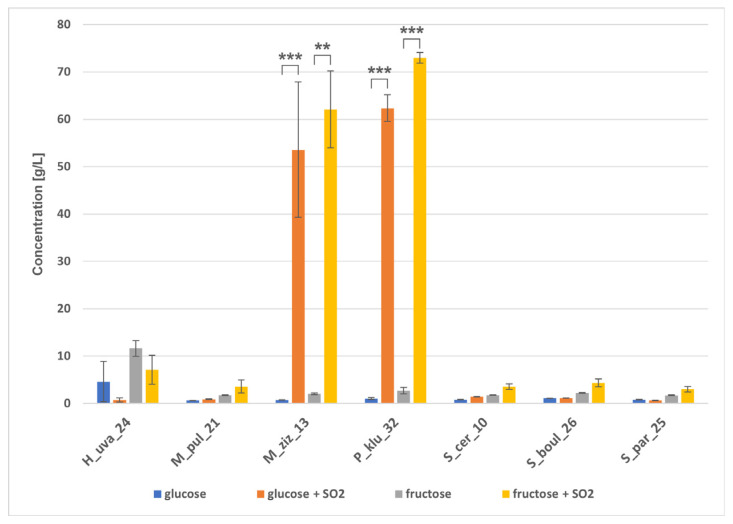
Residual glucose and fructose concentrations (g/L) after fermentation of grape juice by the indicated yeast strains under two fermentation conditions: without SO_2_ and with SO_2_ addition. Bars represent mean ± SD. Differences between conditions were tested using a paired Student’s *t*-test. Brackets and asterisks indicate statistically significant differences between −SO_2_ and +SO_2_ within each strain (* *p* < 0.05; ** *p* < 0.01; *** *p* < 0.001).

**Figure 9:**
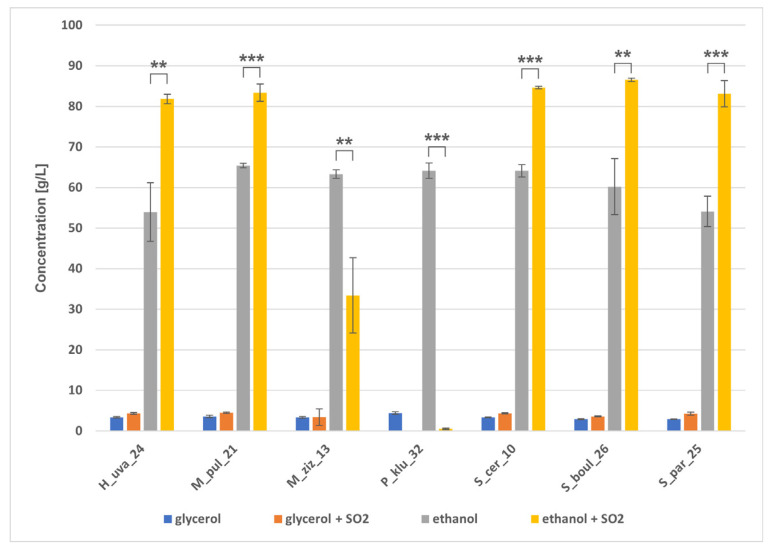
Glycerol and ethanol concentrations (g/L) produced after fermentation of grape juice by the indicated yeast strains under two fermentation conditions: without SO_2_ and with SO_2_ addition. Bars represent mean ± SD. Differences between conditions were tested using a paired Student’s *t*-test. Brackets and asterisks indicate statistically significant differences between −SO_2_ and +SO_2_ within each strain (* *p* < 0.05; ** *p* < 0.01; *** *p* < 0.001).

**Figure 10:**
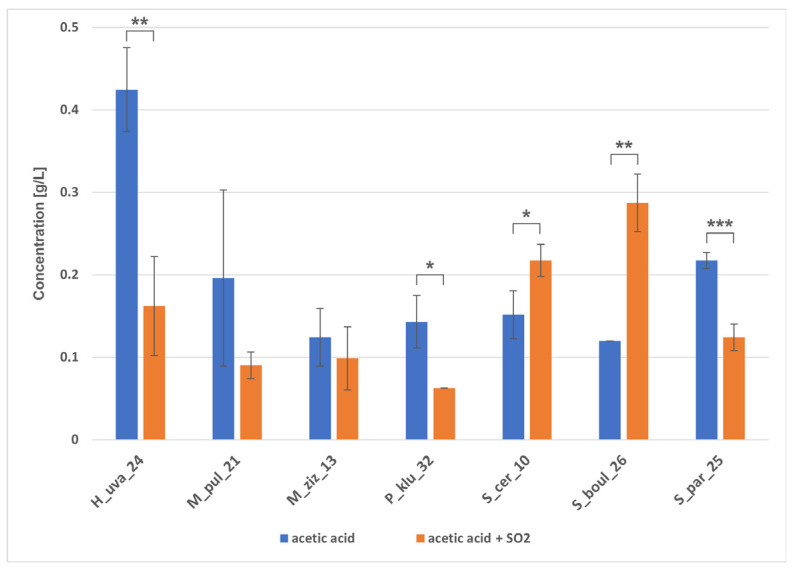
Acetic acid concentration (g/L) produced after fermentation of grape juice by the indicated yeast strains under two fermentation conditions: without SO_2_ and with SO_2_ addition. Bars represent mean ± SD. Differences between conditions were tested using a paired Student’s *t*-test. Brackets and asterisks indicate statistically significant differences between −SO_2_ and +SO_2_ within each strain (* *p* < 0.05; ** *p* < 0.01; *** *p* < 0.001).

HPLC analysis of fermented grape juice samples confirmed the trend towards glucose and fructose fermentation within 10 days (Figure 8). The slowest consumption of sugars was observed in H_uva_24 in juice without SO_2_, and in sulphurised juice by P_klu_32, followed by M_ziz_13. During grape juice fermentation, all strains produced ethanol in the range of 53.9–65.4 g/L (Figure 9). Non-*Saccharomyces* yeasts showed relatively high ethanol production performance, while *Saccharomyces* strains did not seem to reach their optimal potential, producing ethanol concentrations comparable to some non-*Saccharomyces* yeasts. In sulphurised wine, however, all *Saccharomyces* strains produced similar amounts of ethanol, exceeding 83 g/L, while among non-*Saccharomyces* strains, ethanol concentrations varied at different levels of statistical significance, with the lowest ethanol detected in sample P_klu_32 (0.5 g/L). Figure 9 shows that the yeasts had varying abilities to produce glycerol. The level of this polyol ranged from 2.9 g/L (S_par_25, S_boul_26) to 4.4 g/L (P_klu_32) in wines without SO_2_ and from 3.4 g/L (M_ziz_13) to 4.5 g/L (M_pul_21) in sulphurised wines, but a statistically significant difference appeared only in the case of P_klu_32, which did not produce glycerol at all due to poor metabolic activity under these conditions. Each strain in both fermentation variants produced a small amount of acetic acid (Figure 10), and there were visible differences between strains depending on SO_2_, indicating the statistical significance of the interaction between the two factors. In turn, GC/MS analysis did not reveal the presence of ethyl acetate in any of the samples tested. Analysis of the entire fermentation process showed that P_klu_32 was the most sensitive to SO_2_, exhibiting minimal activity under these conditions. The differences in sugar and metabolite concentrations in both variants of the small-scale fermentations also result from the different sugar content in the grapes used for the tests, which was probably related to the ripeness of the fruit harvested at different times. The literature data show differences in the fermentation activity of non-*Saccharomyces* strains, which can produce quite high concentrations of ethanol, e.g., in the study by Mendoza et al. [35]. *H. uvarum* produced 4.84–8.52% *v*/*v* ethanol, *M. pulcherrima* 7.15–7.76%, but *S. cerevisiae* 12–13% (*v*/*v*). Similarly, 41.24–67.47 g/L of sugars remained in 18 strains of *H. uvarum*, and 52.76–56.38 g/L of sugars remained in *Metschnikowia* strains, while *S. cerevisiae* consumed almost all of the sugars. In a study by Lai et al. [52], the authors reported that *H. uvarum* Pi235, *P. kluyveri* Pe114, and *H. guilliermondii* Ki135 produced 83.01, 53.53, and 78.98 g/L of ethanol, respectively, over 7 days, and sugar concentrations were still high (55.12, 107.00, and 57.91 g/L, respectively). In Akan et al. [53], *H. uvarum* B079 and *P. kudriavzevii* B143 produced 79.7 g/L and 66.7 g/L of ethanol, respectively, whilst the *S. cerevisiae* isolate EC1118 produced only 77.7 g/L. The results of ethanol fermentation capacity presented here show that some non-*Saccharomyces* strains are capable of producing ethanol at a level comparable to that of *S. cerevisiae*. It is difficult to explain this phenomenon, but these unexpected results were sometimes obtained, as shown above, and are explained as the response of yeast cells to specific conditions prevailing during fermentation, related to sugar composition, oxygen availability, and temperature [52], rather than metabolic variability of the strains. In turn, the amount of acetic acid in *H. uvarum* was approx. 1.18 g/L, and the authors pointed out that the content of this acid at a level of 0.45–0.7 g/L is an acceptable concentration in wines [35] and a value > 0.8 g/L can be considered harmful [8]. In contrast, in studies by Tofalo et al. [34], during wine fermentation, *Hanseniaspora*, *Metschnilowia* and *Pichia* strains produced approximately 0.3 g/L of citric acid, 1–1.6 g/L of malic acid, 0.16–0.9 g/L of acetic acid and 4.8–5.7 g/L of tartaric acid. Some researchers [54] claimed that native yeast strains operating in a specific environment are not always capable of efficient ethanol fermentation, which may explain the poorer results of our *Saccharomyces* strains. In turn, high acetic acid production is explained by high sugar concentrations inducing osmotic stress, which promotes the accumulation of acetic acid [54]. Other studies report that the fermentation performance of *M. pulcherrima* is assessed differently, with many strains producing up to 4% (*v*/*v*) ethanol, but there are also reports of production of up to 6–7% (*v*/*v*) [55]. To summarise the fermentation capabilities of the isolates obtained, there are clear similarities in the profiles of sugar utilisation, ethanol production as the main metabolite and glycerol as a co-metabolite. The effect of sulphurisation on the slowing down of the metabolism of some yeasts, especially P_klu_32, is evident.

#### 2.6.4. Volatile Compounds

Thirty-two compounds belonging to several groups were identified in small-scale fermentations carried out by *Saccharomyces* and non-*Saccharomyces* yeast: esters (11 compounds), alcohols (11 compounds), acids (five compounds), ketones (two compounds), sulfur compounds (one compound), furans (one compound) and volatile phenols (one compound) (Table 5). Table 5 shows the concentrations of volatile compounds in small-scale fermentations with and without the addition of potassium metabisulfite and statistical analysis results are presented in Supplementary Materials (Supplementary S4).

The most abundant esters in wines performed by *Saccharomyces* and non-*Saccharomyces* yeasts, without or with the addition of potassium metabisulfite, were 2-phenylethyl acetate and ethyl decanoate, with the exception of the *Pichia kluyveri* small-scale fermentations with the addition of potassium metabisulfite. These samples revealed the presence of only one ester: methyl hexadecanoate. Phenylethyl acetate and ethyl decanoate are responsible for the fruity and floral aromas [56]. 3-Methylbutan-1-ol and 2-phenylethanol were the main alcohols present in the small-scale fermentations, with the exception of the M_ziz_13 fermentation with SO_2_ addition. 3-Methylbutan-1-ol was present at the highest concentration in the S_boul_26 fermentation samples without SO_2_ addition, and was absent in the M_ziz_13 small-scale fermentation with SO_2_ addition. Moderate concentrations of 3-methyl-1-butanol and 2-phenylethanol can have a positive impact on wine, providing floral and fruity aromas [57]. Similarly, 3-methylbutan-1-ol and 2-phenylethanol were the main alcohols identified by many authors in studies involving endogenous strains of *H. uvarum* [58], *S. cerevisiae* [59,60], *M. pulcherrima* [61] and a combination of *M. pulcherrima* and *S. cerevisiae* [62]. In most small-scale fermentations with or without the addition of potassium metabisulfite, performed by *Saccharomyces* and non-*Saccharomyces* yeasts, the dominant acids were decanoic and octanoic acids. Furthermore, acetic acid dominated in several small-scale fermentations, e.g., M_pul_21 without added SO_2_, M_ziz_13 and S_cer_10 with and without added SO_2_. Alves et al. [63] found that the most dominant acids for indigenous *S. cerevisiae* strains were octanoic acid, decanoic acid and acetic acid. Acetic acid is responsible for 90% of the volatile acidity of wines and gives them a rancid and pungent odour [61]. The main volatile compound in the ketone class was 4-methyl-3-penten-2-one. One compound was determined in each of the sulfur compound, furan compound and volatile phenol classes.

Statistically significant differences were found in the contents of 2-ethylhexan-1-ol, decanoic acid and octanoic acid among all volatile compounds determined in microvinifications conducted with *Saccharomyces* and non-*Saccharomyces* yeasts. The content of these compounds in samples with *Saccharomyces* yeast and SO_2_ addition was significantly higher. 2-Ethylhexan-1-ol has a citrus and floral aroma, often associated with the scent of roses, as well as oily and fresh notes [64]. According to Carpena et al. [57], octanoic acid and decanoic acid can release unpleasant aromas. Generally, statistically significant differences in volatile compounds were not observed between small-scale fermentations with different yeast strains, with or without the addition of SO_2_. However, when each strain was considered separately, it was found that many compounds differed significantly between fermentations with and without SO_2_ addition. The content of volatile compounds in wine samples without SO_2_ addition was higher for most of these compounds. For example, for M_pul_21, 14 out of 32 compounds had higher values without SO_2_ addition; for S_par_25, it was 18 and 12; for H_uva_24, 14 and 12; for S_boul_26, 23 and 21; for P_klu_32, 13 and 10; for M_ziz_13, 17 and 15; and for S_cer_10, 20 and 16. According to Silva and van Wyk [65], the content of volatile compounds in wines with and without added SO_2_ differs because SO_2_ affects the microbiological and chemical processes occurring during fermentation. The absence of sulfites allows a wider range of yeasts and bacteria to develop and accelerates oxidation processes, thereby changing the aromatic profile of wine. Several strains (e.g., H_uva_24) showed more than 6-fold differences in the content of some volatile compounds with and without SO_2_ treatment. Sulfur dioxide causes a number of cellular changes, such as a modification of membrane transport activity by binding to membrane proteins, the inhibition of glyceraldehyde-3-phosphate dehydrogenase (GAPDH)—an enzyme in the glycolysis pathway and other enzymes such as NAD-glutamate dehydrogenase, alcohol dehydrogenase and ATPase, resulting in a decrease in the ATP content in cells, the modification of the expression of many genes correlated with cell metabolism, the degradation of available thiamine, the binding of metabolites (acetaldehyde, glucose, pyruvate, dihydroxyacetone-phosphate, α-ketoglutaric acid and oxaloacetic acid), thus preventing their further use as substrates in metabolic pathways [66]. Within the alcohol class, small-scale fermentations using *H. uvarum* and no SO_2_ contained significantly higher levels of 2-phenylethyl acetate than fermentations using other yeast strains and no SO_2_. All samples using non-*Saccharomyces* yeasts and no SO_2_ differed significantly in their content of 2-phenylethyl acetate. S_cer_10 produced significantly more of this ester than S_par_25 and S_boul_26, which produced the same amount. No significant differences in 2-phenylethyl acetate production were observed in small-scale fermentations with the non-*Saccharomyces* yeast P_klu_32 containing SO_2_ or with the *Saccharomyces* yeast S_cer_10. Similarly, no significant differences were observed between the fermentations M_ziz_13, S_boul_26 and S_par_25. The content of this compound was significantly higher in all small-scale fermentations without SO_2_ addition than with it, except for the fermentation with M_pul_21, where no statistically significant differences were observed. The differences in the content of ethyl decanoate and ethyl dodecanoate involving both *Saccharomyces* and non-*Saccharomyces* yeast strains with SO_2_ addition were statistically insignificant. Detailed data on differences in the content of individual volatile compounds are provided in Table 5, where statistical differences are marked with different letters.

## 3. Materials and Methods

### 3.1. Microorganisms

Yeast isolates were obtained from wines produced from Regent variety grapes during spontaneous fermentation, as described by Kordowska-Wiater et al. [19]. The grapes of this popular variety in Poland [1] are sourced from three vineyards: “Dom Bliskowice” (DB) (50°56′58.2″ N 21°52′16.6″ E), “Małe Dobre” (MD) (51°17′50.2″ N 21°56′26.7″ E), and “Winnica Janowiec” (WJ) (51°19′19.1″ N 21°53′36.4″ E) located in the Małopolska Vistula Gorge region. Yeasts were isolated according to Section 3.2., and genetically identified acc. to method described by Staniszewski and Kordowska-Wiater [20]. Seven selected strains, representing various species, presented in Table 6, are used for testing. The initial criterion for selecting isolates was their growth in YPG medium after ten passages. Strains that showed no decline in growth were selected for the next stage, in which one representative of each species was selected, based on the fastest growth rate among strains belonging to the same species 

Yeasts used for the killer activity study: *Kluyveromyces lactis* Y-6682 was obtained from NRRL (ARS Culture and Patent Culture Collections, US Department of Agriculture, Peoria, IL, USA), *Candida parapsilosis* C29 and *C. freyschussii* C16 were obtained from the culture collection of the Department of Biotechnology, Microbiology and Human Nutrition, University of Life Sciences in Lublin, Poland. These strains were recommended as sensitive by Wójcik and Kordowska-Wiater [42].

Yeast cultures were stored frozen in 30% glycerol at −80 °C, working cultures were stored on YPD (Yeast Extract Peptone Dextrose) (BTL, Łódź, Poland) agar slants at +4 °C, and before experiments, they were cultured for 48 h at 28 °C in YPD broth.

### 3.2. Isolation of Yeasts from Wines and Pure Cultures Obtaining

During the spontaneous fermentation of grapes described in the publication by Kordowska-Wiater et al. [19], wine samples were taken sterilely under anaerobic conditions at different stages (every two days) until fermentation stopped. Serial dilutions of wine samples in the range of 10^−1^ to 10^−5^ were made, and 0.1 mL was spread on Petri dishes with medium dedicated for yeast isolation -YGC (yeast extract, glucose, chloramphenicol) agar (BTL, Łódź, Poland). The plates were incubated for 3–5 days at 25 °C. The typical singular colonies of yeasts were seeded onto WL Nutrient Agar (Oxoid, Basingstoke, UK) to presumptively discriminate the yeast species by colony morphology and colour [7]. After incubation at 25 °C for the next 3 days, the various colony types were isolated and maintained on YPG slants at 4 °C. Some passages and microscopic observations (Microscope Olympus C-23, Evident, Tokyo, Japan) were performed to obtain and confirm pure cultures. The strains confirmed were preserved in freezing conditions (−80 °C) with glycerol (30%).

### 3.3. The Genetic Identification

The genetic identification of yeast strains was based on PCR of the ITS1-5.8S rDNA-ITS2 region using primers ITS1 (5′TCCGTAGGTGAACCTGCGG-3′) and ITS4 (5′-TCCTCCGCTTATTGATATGC-3′) and PCR Mix Plus Green (A&A Biotechnology, Gdańsk, Poland). The amplicons obtained were sequenced with the Sanger method (Genomed, Warsaw, Poland) and bioinformatically analysed as described in detail by Staniszewski and Kordowska-Wiater [20].

### 3.4. Enological Characteristics of Selected Yeasts

#### 3.4.1. Ethanol Tolerance

It was determined in liquid YPG medium supplemented with 96% *v*/*v* ethanol to obtain finally 2, 4, 6, 8, 10, 12, 14 and 16% *v*/*v* ethanol in the medium. YPG without ethanol was used as a control medium. The inocula of yeasts were prepared in YPG medium without the addition of ethanol after incubation at 28 °C for 24 h. They were used for the inoculation of test and control tubes. The following variants were prepared based on scientific literature about yeast resistance to ethanol: *M. pulcherrima* and *M. ziziphicola* strains were added to the media containing 2, 4, 6, 8 and 10% *v*/*v* ethanol; *H. uwarum* and *P. kluyveri* were added to the media with 2, 4, 6, 8, 10 and 12% *v*/*v* ethanol and three *Saccharomyces* strains were added to the media with 8, 10, 12, 14 and 16% *v*/*v* ethanol. Each culture variant was performed in three replicates. All samples were incubated at 28 °C for 48 h and measurement of optical density at 600 nm (*OD*600) was performed after 24 and 48 h on Smart Spec Plus spectrophotometer (Bio-Rad Laboratories, Hercules, CA, USA). These measurements were used for the calculation of the yeast relative growth (RG), which is an indicator of the yeast’s response to the presence of a stressor, manifested as a slowing of the growth rate, according to the formula:

(1) $$\mathrm{RG}\;=\;\frac{OD600\;sample}{OD600\;control}\times 100\%$$

#### 3.4.2. Enzymatic Activity

The enzymatic activities of the strains were determined with the semiquantitative API ZYM system (bio-Merieux, Craponne, France) following the manufacturer’s instructions. Cell suspensions of fresh cultures (5 in the McFarland standard) were transferred into the wells of the API ZYM strips and incubated for 4 h at 28 °C. The developing reagents ZYM A and ZYM B were added to each well after incubation to observe colour changes, indicating positive reactions. The evaluation was made on the basis of the API ZYM colour chart [67].

Pectinolytic activity was determined in the medium composed of lemon pectin (2 g/L), yeast extract (1 g/L), agar (15 g/L), KH_2_PO_4_ (0.2 g/L), CaCl_2_ (0.05 g/L), (NH_4_)_2_SO_4_ (1 g/L), MgSO_4_ × 7 H_2_O (0.8 g/L), MnSO_4_ (0.05 g/L), pH 4.5. The medium was inoculated with the tested strains using a microbiological loop and incubated at 25 °C for 48 h. After incubation, the plates were flooded with Lugol’s solution and brightening zones around the colonies after 10 min indicated pectolytic activity.

#### 3.4.3. H_2_S Production

BIGGY Agar (Biomaxima, Lublin, Poland) on Petri dishes was inoculated with individual yeast species. After incubation at 25 °C for 48 h, the results were read by observing the colour of the colonies in brown and black. The colour intensity was assessed on a scale from 0 to 3, where 0 means no colour (no H_2_S) and 3 means brown–black colour (large amount of H_2_S) [68].

### 3.5. Killer Activity

The experiment was carried out using the modified well-diffusion method described by Mendoza et al. [35]. The YPG agar with methylene blue (0.003% *w*/*v*) and the YPG agar with methylene blue and 2% NaCl, which often stimulates the production of killer proteins, were used. Liquid cultures (100 μL) of sensitive, reference strains—*K. lactis*, *C. parapsilosis*, *C. freyschussii*—were spread on the agar medium. Then, 100 μL of the tested yeast suspensions were introduced into wells in two repetitions. The plates were incubated at 20 °C for 48 h. After incubation, the formation of growth inhibition zones of sensitive strains, surrounded by a blue border around the wells, was checked [35].

### 3.6. Small-Scale Fermentations

#### 3.6.1. Small-Scale Fermentations of Chemically Defined Grape Juice Medium (CDGJM) by Monocultures

CDGJM acc. to Jiranek and Henschke [44], at pH 3.5, was sterilised by filtration and 100 mL were poured into sterile conical flasks equipped with stoppers with silent fermentation tubes. An inoculum of each tested yeast was prepared in 10 mL of liquid YPG medium. The cultures were incubated for 48 h at 25 °C, then centrifuged for 10 min (8000 rpm), washed and suspended in sterile distilled water. Two mL of inoculum were added to 100 mL of CDGJM. The initial yeast population in each flask was determined using the plate count method described in Section 3.7.3. The flasks were incubated at 25 °C for 15 days under static conditions. These fermentations as biological replicates were performed in triplicate.

#### 3.6.2. Small-Scale Fermentations of Grape Juice by Monocultures

Pasteurised juice obtained from Regent variety grapes was centrifuged at 6000 rpm in a Sorvall Legend Xfr centrifuge (Thermo Scientific, Waltham, MA, USA) for 15 min. After pouring 100 mL of juice into sterile conical flasks equipped with stoppers and silent fermentation tubes, they were inoculated in the same way as described in Section 3.6.1. For a separate set of experiment 0.01 g of potassium metabisulphate dissolved in 0.2 mL of grape juice was added to each sample, intended to test the sensitivity of yeast to SO_2_. The initial yeast numbers in the fermentation batches were determined according to Section 3.7.3. The flasks were incubated at 25 °C for 10 days under static conditions. These fermentations as biological replicates were performed in triplicate.

### 3.7. Analysis of Fermentation

#### 3.7.1. Rate of Fermentation

For the determination of fermentation rate, the flasks were weighed every day at a fixed time and the weight loss corresponding to the amount of released CO_2_ was calculated.

#### 3.7.2. pH

The pH values were determined using a pH metre HI2210-02 (Hanna Instruments, Olsztyn, Poland).

#### 3.7.3. The Growth of Yeasts

After inoculating the CDGJM and grape juice with yeast according to Section 3.6.1, all flasks were agitated, and 0.1 mL of each sample was taken for tenfold dilutions preparing. Serial dilutions in the range of 10^−1^ to 10^−5^ were made and 0.1 mL was spread on Petri dishes with YPG agar. The plates were incubated for two days at 25 °C. Then, after fermentation time, 0.1 mL samples were taken again from the wine flasks after agitation, tenfold dilutions were made, and 0.1 mL of the dilutions was spread on YPG plates. The plates were incubated as above. The colonies were counted and their number was calculated.

#### 3.7.4. HPLC Analysis

The samples taken from all the flasks after fermentation process were filtered using 0.22 µm pore size PTFE membrane syringe filters, diluted with distilled water, and then analysed using a high-performance liquid chromatography system Dionex UltiMate 3000 (Thermo Scientific, Waltham, MA, USA) comprising a pump (LPG-3400SD), an autosampler (WPS-3000SL), a column oven (TCC-3000SD), and Aminex HPX-87H column (300 × 7.8 mm, Bio-Rad Laboratories, Hercules, CA, USA). A 3 mM H_2_SO_4_ aqueous solution was used as the mobile phase with a flow rate of 0.5 mL/min. The analysis was conducted to detect sugars, glycerol and ethanol using an RI detector (RefractoMax 521, Thermo Scientific, Waltham, MA, USA), and organic acids using a UV-Vis detector (Dionex Ultimate, Thermo Scientific, Waltham, MA, USA). The methodological details were described by Staniszewski et al. [27].

#### 3.7.5. GC/MS Analysis

The analysis of ethanol and ethyl acetate in the wine samples after deproteinisation was carried out using a gas chromatograph/mass spectrometer (GC/MS) (Model GC2010, Shimadzu, Kyoto, Japan) coupled with an MS-EI apparatus (model QP 2010Plus, Shimadzu, Japan) and an auto injector (model AOC-20i, Shimadzu, Japan). CP-WAX 57 CB (Agilent J&W, Santa Clara, CA, USA) column operated in the splitless mode with the valve closed for 0.3 min. Helium was used as a carrier gas. Injector and detector temperature was 200 °C. Details of the analysis were published by Kordowska-Wiater et al. [69].

#### 3.7.6. Analysis of Volatile Compounds

Volatile compounds produced during small-scale fermentations of grape juice by individual yeast strains were determined using the SPME method coupled with GC/MS detection. A fibre for the extraction of wines was PA (Supelco, Bellefonte, PA, USA). VF-WAXms capillary column (60 m, 0.25 mmID × 0.25 µm film thickness and 100% polyethylene glycol) (Agilent, Santa Clara, CA, USA) was used for separation. Helium as a carrier gas was used at a flow rate of 1.8 mL/min. Other details are described by Stój et al. [70].

### 3.8. Statistical Analysis

The results (means and standard deviations) of the amount of CO_2_ released, the number of yeast cells, and pH changes during small-scale fermentations were calculated using Excel for Microsoft Office 365. Statistical analysis of the obtained results of OD600 measurement, HPLC and GC/MS was carried out using Statistica ver. 13.3 (2017) for Windows (StatSoft Inc., Tulsa, OK, USA). A one-way analysis of variance (ANOVA) was used to compare the production and consumption of the analysed compounds by the tested yeast isolates in samples obtained from CDGJM fermentation. A two-way ANOVA was applied to identify statistically significant differences in the consumption of sugars, production of alcohols, acetic acid, and volatile compounds after grape juice fermentation by individual yeast isolates, depending on whether SO_2_ was added or not. A two-way ANOVA was also applied to compare the relative growth of yeast strains as an indicator of ethanol tolerance depending on ethanol concentration and time. The significance of differences between mean values was evaluated using Tukey’s post hoc test, with the level of statistical significance set at *p* < 0.01.

## 4. Conclusions

Spontaneously fermented grape wines are a suitable environment for yeast growth, resulting in the isolation of 44 strains belonging to four genera. The isolates selected for the study were genetically identified by Sanger sequencing of the ITS1-5.8S-ITS4 rDNA region as follows: *S. cerevisiae*, *S. paradoxus*, *S. cerevisiae* var. *boulardii*, *M. pulcherrima*, *M. ziziphicola*, *H. uvarum* and *P. kluyveri*. The results obtained during the analysis of the oenological characteristics of these yeasts revealed that they have different properties in terms of ethanol tolerance, H_2_S production, and enzymes important for winemaking, such as β-glucosidase, but not pectinase. The lack of killer activity makes it possible to combine strains and conduct fermentation in standardised mixed cultures, which is a starting point for further research on improving the properties of wines produced in the cool climate of Poland. The fermentation performance determined in the original grape juice proved satisfactory in terms of ethanol and volatile compound concentrations. The results of the study indicate that the non-*Saccharomyces* yeast isolates exhibited a high capacity for ethanol production in grape juice, even exceeding 60 g/L (with the exception of H_uva_24). M_pul_21 and H_uva_24 were resistant to SO_2_ in grape juice, producing even over 80 g/L of ethanol, which may have been caused by specific conditions prevailing during fermentation. Taking into account significant enzymatic activities, high levels of β-glucosidase and α-glucosidase activity were demonstrated in the genus Metschnikowia: M_pul_21, M_ziz_13 and Pichia–P_klu_32. All non-*Saccharomyces* strains produced relatively large amounts of volatile compounds responsible for floral and fruity aromas. In contrast, P_klu_32 and M_ziz_13 are not suitable for use in sulphurised musts, which may be a disqualifying factor. Three *Saccharomyces* strains exhibited similar characteristics typical of the genus; they grew well and produced ethanol in sulphurised musts, but in the presence of SO_2_ they produced significantly fewer volatile aromatic compounds. They did not produce β-glucosidase, and only S_boul_26 exhibited α-glucosidase activity. The above studies are in line with the global trend in recent years of searching for native yeast strains associated with a given wine region, which can be used as starter monocultures. Their ability to ferment ethanol effectively or moderately and simultaneously produce characteristic volatile compounds can also be considered a positive feature in terms of obtaining beverages with reduced alcohol content and interesting aromatic profiles, which is one of the current trends in the alcoholic beverage industry.

## Figures and Tables

**Table 1:** Identification of yeast isolates, the number of isolates, and the characteristics of the colonies.

Species	No. of Isolates	Morphology of Colonies on WL Nutrient Agar
*Saccharomyces cerevisiae*	11	White, regular, round, convex, diameter 1–2 to 5–6 mm
*Saccharomyces cerevisiae*/*paradoxus*	1	White, regular, round, convex, diameter 5 mm
*Saccharomyces cerevisiae* var. *boulardii*	1	White, regular, round, convex, diameter 5 mm
*Metchnikowia pulcherrima*	6	Regular, smooth, round, white or white-green top, orange underneath, diameter 1–5 mm
*Metschnikowia ziziphicola*	1	white top, greenish bottom, diameter 3–5 mm
*Metschnikowia* sp. *	1	dirty white top, dark bottom, diameter 1–2 mm
*Pichia kluyveri*	2	dirty green or dark green, wavy, irregular, spreading out diameter above 5 mm
*Hanseniaspora uvarum*	10	light or dark green, round, smooth, flat, diameter 3–5 mm
*Starmerella bacillaris* *	11	white, regular, small, 1–3 mm

* *Metschnikowia* sp. and *Starmerella bacillaris* isolates were excluded from further testing due to very poor growth in laboratory conditions.

**Table 2:** Enzyme activities of the selected yeast isolates were determined using the API-ZYM system.

Enzyme	Isolate						
	10	13	21	24	25	26	32
Alkaline phosphatase	5	2	2	4	5	5	5
Esterase (C4)	4	4	4	0	3	3	4
Esterase–lipase (C8)	3	3	2	0	2	1	2
Lipase (C14)	1	0	0	0	0	0	0
Leucine arylamidase	5	5	5	1	5	5	5
Valine arylamidase	4	4	4	1	4	4	3
Cystine arylamidase	3	1	1	0	3	3	0
Trypsin	0	0	0	0	0	0	0
α-Chymotrypsin	0	0	0	0	0	0	0
Acid phosphatase	5	4	5	5	5	5	5
Naphthol-AS-BI-phosphohydrolase	5	3	3	3	5	5	4
α-Galactosidase	0	0	0	0	0	0	0
β-Galactosidase	0	0	0	0	0	0	0
β-Glucuronidase	0	0	0	0	0	0	0
α-Glucosidase	1	5	5	0	1	5	3
β-Glucosidase	0	4	3	0	1	0	3
N-acetyl-β-glucosaminidase	0	0	0	0	0	0	0
α-Mannosidase	1	0	0	0	1	2	2
α-Fucosidase	0	0	0	0	0	0	0

0–1—no enzymatic activity; 2–3—weak activity; 4–5—strong activity.

**Table 3:** The number of yeast cells in CDGJ medium [mean cfu/mL] and pH after fermentation [means, *n* = 3].

Time [Days]	S_cer_10	S_par_25	S_boul_26	M_ziz_13	M_pul_21	H_uva_24	P_klu_32
0	3.3 × 10^5^	1.25 × 10^5^	1.0 × 10^5^	1.35 × 10^5^	1.75 × 10^5^	1.45 × 10^5^	2.15 × 10^5^
15	2.63 × 10^4^	3.58 × 10^4^	1.21 × 10^4^	1.33 × 10^6^	1.2 × 10^5^	2.0 × 10^5^	1.03 × 10^6^
pH	3.31	3.37	3.36	3.42	3.44	3.34	3.39

**Table 4:** The number of yeast cells in grape juice without and with SO_2_ [mean cfu/mL] and pH after fermentation [means, *n* = 3].

Time [Days]	S_cer_10	S_par_25	S_boul_26	M_ziz_13	M_pul_21	H_uva_24	P_klu_32
Grape juice without SO_2_							
0	4.85 × 10^5^	4.8 × 10^5^	7.35 × 10^5^	5.45 × 10^5^	1.55 × 10^5^	1.3 × 10^5^	2.74 × 10^6^
10	7.02 × 10^8^	6.84 × 10^8^	1.71 × 10^8^	1.09 × 10^9^	7.98 × 10^8^	1.25 × 10^9^	1.5 × 10^9^
pH	3.62	3.68	3.67	3.68	3.67	3.7	3.61
Grape juice with SO_2_							
0	1.48 × 10^6^	1.36 × 10^6^	9.75 × 10^5^	6.6 × 10^5^	1.0 × 10^5^	2.15 × 10^5^	1.43 × 10^6^
10	2.85 × 10^8^	7.16 × 10^8^	8.65 × 10^8^	6.49 × 10^8^	6.4 × 10^8^	8.24 × 10^8^	5.0 × 10^5^
pH	3.15	3.12	3.15	3.15	3.1	3.21	3.2

**Table 5:** Mean concentrations of volatile compounds produced in monocultures of different yeast strains grown on grape juice under SO_2_-treated and untreated conditions.

Compounds	Yeast Strains													
	M_pul_21		S_par_25		H_uva_24		S_boul_26		P_klu_32		M_ziz_13		S_cer_10	
	No SO_2_	SO_2_	No SO_2_	SO_2_	No SO_2_	SO_2_	No SO_2_	SO_2_	No SO_2_	SO_2_	No SO_2_	SO_2_	No SO_2_	SO_2_
Esters														
*2-Phenylethyl acetate*	38.18 ^d^	30.90 ^d^	131.89 ^c^	45.46 ^d^	927.52 ^a^	153.44 ^c^	130.76 ^c^	7.96 ^d^	251.01 ^b^	0.00 ^d^	127.43 ^c^	14.10 ^d^	228.38 ^b^	32.40 ^d^
*3-Methyl-1-butyl acetate*	11.44 ^de^	11.89 ^de^	42.75 ^c^	34.40 ^cd^	114.36 ^a^	10.59 ^de^	86.22 ^b^	3.44 ^e^	7.76 ^e^	0.00 ^e^	101.45 ^ab^	4.48 ^e^	44.99 ^c^	9.10 ^de^
*Diethyl butanedioate*	3.76 ^abc^	0.00 ^e^	3.39 ^bcd^	0.00 ^e^	0.70 ^cde^	0.00 ^e^	3.95 ^ab^	0.00 ^e^	0.51 ^de^	0.00 ^e^	0.00 ^e^	0.65 ^cde^	6.79 ^a^	0.00 ^e^
*Ethyl 2-hydroxypropanoate*	0.00 ^c^	0.00 ^c^	0.00 ^c^	0.00 ^c^	0.00 ^c^	0.00 ^c^	0.00 ^c^	0.00 ^c^	4.39 ^a^	0.00 ^c^	0.00 ^c^	0.00 ^c^	0.00 ^c^	2.08 ^b^
*Ethyl 9-decenoate*	0.00 ^b^	0.00 ^b^	0.00 ^b^	0.00 ^b^	1.89 ^a^	0.49 ^b^	0.00 ^b^	0.00 ^b^	2.52 ^a^	0.00 ^b^	0.00 ^b^	0.00 ^b^	0.00 ^b^	0.00 ^b^
*Ethyl decanoate*	85.11 ^def^	64.12 ^def^	240.43 ^c^	94.56 ^de^	139.56 ^d^	54.32 ^def^	623.83 ^b^	23.08 ^ef^	23.06 ^ef^	0.00 ^f^	625.09 ^b^	47.25 ^ef^	715.30 ^a^	62.11 ^def^
*Ethyl dodecanoate*	0.00 ^d^	31.84 ^d^	189.62 ^bc^	77.89 ^cd^	67.69 ^d^	41.68 ^d^	447.83 ^a^	9.57 ^d^	0.00 ^d^	0.00 ^d^	116.37 ^bcd^	0.00 ^d^	231.35 ^b^	0.00 ^d^
*Ethyl hexadec-9-enoate*	0.00 ^d^	1.77 ^d^	5.98 ^bcd^	17.43 ^a^	5.47 ^cd^	2.92 ^d^	7.02 ^bcd^	3.48 ^d^	2.89 ^d^	0.00 ^d^	0.00 ^d^	0.00 ^d^	12.91 ^abc^	14.59 ^ab^
*Ethyl hexanoate*	9.65 ^de^	10.42 ^de^	39.27 ^bc^	33.26 ^bc^	22.86 ^cd^	8.71 ^de^	40.76 ^bc^	0.00 ^e^	5.65 ^de^	0.00 ^e^	74.90 ^a^	3.60 ^de^	53.98 ^ab^	9.98 ^de^
*Ethyl octanoate*	13.10 ^cd^	4.29 ^cd^	44.31 ^b^	0.00 ^d^	50.00 ^b^	11.52 ^cd^	122.63 ^a^	7.99 ^cd^	29.53 ^bc^	0.00 ^d^	0.00 ^d^	1.07 ^d^	97.39 ^a^	0.00 ^d^
*Methyl hexadecanoate*	0.00 ^e^	1.01 ^de^	0.00 ^e^	6.80 ^b^	0.00 ^e^	1.36 ^de^	4.83 ^bc^	2.05 ^de^	1.30 ^de^	10.05 ^a^	0.00 ^e^	2.48 ^d^	0.00 ^e^	3.17 ^cd^
Alcohols														
*1-Propanol*	43.88 ^cd^	46.40 ^cd^	38.68 ^cd^	47.09 ^cd^	199.57 ^b^	51.39 ^cd^	38.01 ^cd^	12.35 ^ef^	11.07 ^ef^	0.00 ^f^	315.20 ^a^	11.67 ^ef^	60.64 ^c^	32.70 ^de^
*2-(2-Ethoxyethoxy)- ethanol*	243.08 ^bc^	0.00 ^f^	105.88 ^de^	0.00 ^f^	167.52 ^cd^	0.00 ^f^	351.92 ^a^	0.00 ^f^	70.38 ^ef^	0.00 ^f^	312.30 ^ab^	0.00 ^f^	293.12 ^ab^	318.26 ^ab^
*2-Ethylhexan-1-ol*	17.83 ^d^	1.39 ^e^	67.41 ^b^	0.00 ^e^	6.37 ^e^	0.85 ^e^	66.87 ^b^	1.47 ^e^	17.11 ^d^	0.00 ^e^	58.38 ^c^	1.11 ^e^	100.04 ^a^	2.83 ^e^
*2-Methylpropan-1-ol*	38.77 ^cd^	35.07 ^cde^	35.33 ^cde^	40.38 ^cd^	36.79 ^cde^	26.59 ^def^	90.53 ^b^	28.39 ^def^	88.06 ^b^	0.00 ^g^	154.46 ^a^	18.59 ^efg^	53.18 ^c^	12.75 ^fg^
*2-Phenylethanol*	210.24 ^de^	101.72 ^fg^	268.40 ^cd^	208.75 ^de^	215.83 ^de^	62.87 ^g^	377.96 ^b^	50.79 ^g^	69.32 ^g^	11.44 ^g^	315.96 ^bc^	68.21 ^g^	556.64 ^a^	162.99 ^ef^
*3-Methylbutan-1-ol*	587.24 ^bc^	320.07 ^d^	494.93 ^c^	608.70 ^b^	312.72 ^d^	213.88 ^de^	915.98 ^a^	277.42 ^d^	233.58 ^de^	3.61 ^f^	0.00 ^f^	148.13 ^e^	548.92 ^bc^	267.88 ^d^
*Butan-1-ol*	10.42 ^b^	7.09 ^bcd^	12.05 ^b^	25.47 ^a^	2.89 ^cd^	5.27 ^bcd^	10.18 ^b^	1.59 ^d^	2.76 ^cd^	9.03 ^bc^	11.60 ^b^	1.61 ^d^	7.37 ^bcd^	8.18 ^bcd^
*Butane-2,3-diol*	12.32 ^bc^	0.00 ^e^	6.95 ^cd^	0.00 ^e^	3.79 ^de^	13.95 ^b^	0.00 ^e^	9.17 ^bcd^	7.95 ^cd^	0.00 ^e^	0.00 ^e^	0.00 ^e^	0.00 ^e^	59.47 ^a^
*Dodecan-1-ol*	0.00 ^c^	0.59 ^b^	0.00 ^c^	0.00 ^c^	0.00 ^c^	1.16 ^a^	0.00 ^c^	0.48 ^b^	0.00 ^c^	0.00 ^c^	0.00 ^c^	0.00 ^c^	0.00 ^c^	0.00 ^c^
*Hexan-1-ol*	10.54 ^bc^	5.34 ^cde^	15.65 ^b^	0.00 ^e^	31.19 ^a^	1.91 ^e^	9.29 ^bcd^	6.73 ^cde^	0.95 ^e^	3.53 ^de^	26.11 ^a^	3.32 ^de^	15.98 ^b^	3.57 ^de^
*Propane-1,2,3-triol*	0.00 ^b^	0.00 ^b^	0.00 ^b^	0.00 ^b^	0.00 ^b^	0.00 ^b^	0.00 ^b^	0.00 ^b^	0.00 ^b^	0.00 ^b^	245.62 ^a^	0.00 ^b^	0.00 ^b^	0.00 ^b^
Acids														
*Acetic acid*	71.66 ^c^	6.47 ^g^	81.92 ^c^	77.55 ^c^	174.72 ^a^	38.37 ^de^	23.91 ^ef^	49.76 ^d^	38.54 ^de^	3.28 ^g^	24.97 ^ef^	9.83 ^fg^	49.55 ^d^	99.92 ^b^
*Decanoic acid*	22.02 ^def^	12.17 ^ef^	151.44 ^b^	27.84 ^def^	36.08 ^de^	9.93 ^ef^	181.72 ^a^	11.03 ^ef^	12.47 ^ef^	1.81 ^f^	62.96 ^c^	4.94 ^f^	159.56 ^ab^	43.61 ^cd^
*Dodecanoic acid*	0.00 ^c^	0.00 ^c^	0.00 ^c^	0.00 ^c^	0.00 ^c^	0.00 ^c^	0.00 ^c^	0.00 ^c^	7.27 ^b^	17.23 ^a^	0.00 ^c^	2.41 ^c^	0.00 ^c^	0.00 ^c^
*Hexanoic acid*	0.00 ^b^	0.00 ^b^	0.00 ^b^	0.00 ^b^	43.3326 ^a^	0.00 ^b^	0.00 ^b^	0.00 ^b^	0.00 ^b^	0.00 ^b^	0.00 ^b^	0.00 ^b^	0.00 ^b^	0.00 ^b^
*Octanoic acid*	77.18 ^def^	41.06 ^efg^	480.04 ^b^	78.38 ^def^	147.52 ^d^	33.42 ^fg^	707.93 ^a^	21.72 ^fg^	53.11 ^efg^	0.00 ^g^	225.21 ^c^	12.00 ^fg^	747.39 ^a^	112.46 ^de^
*Ketons*														
*3-Hydroxybutan-2-one*	0.00 ^c^	2.61 ^b^	0.00 ^c^	0.00 ^c^	10.79 ^a^	1.92 ^bc^	0.00 ^c^	2.74 ^b^	1.46 ^bc^	0.00 ^c^	0.00 ^c^	0.00 ^c^	0.00 ^c^	0.00 ^c^
*4-Methyl-3-penten-2-one*	19.63 ^cde^	20.18 ^cde^	50.95 ^a^	26.69 ^bcd^	32.31 ^bc^	13.16 ^de^	44.09 ^ab^	14.47 ^de^	6.61 ^e^	21.51 ^cde^	42.37 ^ab^	10.37 ^de^	41.89 ^ab^	14.39 ^de^
Sulphur compounds														
*3-(Methylsulfanyl) propan-1-ol*	2.15 ^b^	0.92 ^cd^	0.00 ^f^	2.76 ^a^	0.90 ^cde^	0.62 ^cde^	0.00 ^f^	0.39 ^ef^	1.08 ^c^	0.00 ^f^	0.40 ^def^	0.00 ^f^	0.00 ^f^	0.00 ^f^
Furan compounds														
*Dihydrofuran-2(3H)-one*	0.00 ^c^	0.00 ^c^	52.77 ^a^	41.72 ^b^	0.00 ^c^	0.00 ^c^	0.00 ^c^	0.00 ^c^	0.00 ^c^	0.00 ^c^	0.00 ^c^	0.00 ^c^	36.91 ^b^	0.00 ^c^
Volatile phenols														
*Phenol*	0.00 ^d^	1.06 ^d^	2.27 ^cd^	9.60 ^a^	0.00 ^d^	1.38 ^cd^	9.11 ^ab^	2.26 ^cd^	1.67 ^cd^	2.46 ^bcd^	0.00 ^d^	1.25 ^d^	8.09 ^abc^	5.02 ^abcd^

Values are the means of three biological replicates (*n* = 3). Different superscript letters within a compound indicate significant differences among strain × SO_2_ treatments according to Tukey’s HSD test (α = 0.01).

**Table 6:** Yeasts species and strains used for testing.

Species	Strain Number	Code Name	Vineyard
*Saccharomyces cerevisiae*	10	S_cer_10	DB
*Saccharomyces paradoxus*	25	S_par_25	DB
*Saccharomyces cerevisiae* var. *boulardii*	26	S_boul_26	DB
*Metchnikowia pulcherrima*	21	M_pul_21	MD
*Metschnikowia ziziphicola*	13	M_ziz_13	MD
*Pichia kluyveri*	32	P_klu_32	DB
*Hanseniaspora uvarum*	24	H_uva_24	WJ

## Data Availability

Data is contained within the article or Supplementary Materials.

## Supplementary Materials

The following supporting information can be downloaded at: https://www.mdpi.com/article/10.3390/molecules31081274/s1, Supplementary S1: Tukey tests for Figure 1 and Figure 2; Supplementary S2: Tukey tests for Figure 5, Figure 6 and Figure 7; Supplementary S3: Tukey tests for Figure 8, Figure 9 and Figure 10; Supplementary S4: Tukey tests for Table 5.
